# Characterization of a strong and constitutive promoter from the *Arabidopsis* serine carboxypeptidase-like gene *AtSCPL30* as a potential tool for crop transgenic breeding

**DOI:** 10.1186/s12896-018-0470-x

**Published:** 2018-09-21

**Authors:** Pingping Jiang, Ke Zhang, Zhaohua Ding, Qiuxia He, Wendi Li, Shuangfeng Zhu, Wen Cheng, Kewei Zhang, Kunpeng Li

**Affiliations:** 1 The Key Laboratory of Plant Cell Engineering and Germplasm Innovation, Ministry of Education, School of Life Science, Shandong University, Jinan, Shandong China; 2 Maize Institute of Shandong Academy of Agricultural Sciences, Jinan, Shandong China; 3 Biology Institute of Shandong Academy of Sciences, Jinan, Shandong China

**Keywords:** *AtSCPL30*, *CaMV35S*, Constitutive promoter, GUS analysis, *Nicotiana benthamiana*, Stress treatment

## Abstract

**Background:**

Transgenic technology has become an important technique for crop genetic improvement. The application of well-characterized promoters is essential for developing a vector system for efficient genetic transformation. Therefore, isolation and functional validation of more alternative constitutive promoters to the *CaMV35S* promoter is highly desirable.

**Results:**

In this study, a 2093-bp sequence upstream of the translation initiation codon ATG of *AtSCPL30* was isolated as the full-length promoter (PD1). To characterize the *AtSCPL30* promoter (PD1) and eight 5′ deleted fragments (PD2-PD9) of different lengths were fused with *GUS* to produce the promoter*::GUS* plasmids and were translocated into *Nicotiana benthamiana*. PD1-PD9 could confer strong and constitutive expression of transgenes in almost all tissues and development stages in *Nicotiana benthamiana* transgenic plants. Additionally, PD2-PD7 drove transgene expression consistently over twofold higher than the well-used *CaMV35S* promoter under normal and stress conditions. Among them, PD7 was only 456 bp in length, and its transcriptional activity was comparable to that of PD2-PD6. Moreover, GUS transient assay in the leaves of *Nicotiana benthamiana* revealed that the 162-bp (− 456~ − 295 bp) and 111-bp (− 294~ − 184 bp) fragments from the *AtSCPL30* promoter could increase the transcriptional activity of mini35S up to 16- and 18-fold, respectively.

**Conclusions:**

As a small constitutive strong promoter of plant origin, PD7 has the advantage of biosafety and reduces the probability of transgene silencing compared to the virus-derived *CaMV35S* promoter. PD7 would also be an alternative constitutive promoter to the *CaMV35S* promoter when multigene transformation was performed in the same vector, thereby avoiding the overuse of the *CaMV35S* promoter and allowing for the successful application of transgenic technology. And, the 162- and 111-bp fragments will also be very useful for synthetic promoter design based on their high enhancer activities.

**Electronic supplementary material:**

The online version of this article (10.1186/s12896-018-0470-x) contains supplementary material, which is available to authorized users.

## Background

Transgenic technology has enabled incremental crop improvement by the introduction of specific genes or decreasing the expression of endogenous genes, including herbicide tolerance, insect resistance and tolerance to other stresses, to improve farming and production [1]. The major challenges in the successful application of transgenic technology are the precise control of transgenes or endogenous gene expression [2]. Appropriate promoters are crucial for the regulation of the transgenes expression at desired profile, thereby promoting the successful application of transgenic technology, while the number and type of promoters for plant genetic transformation are still quite limited [2–5]. Therefore, cloning and characterization of various promoters suitable for plant genetic transformation is highly desirable [6, 7].

Strong constitutive promoters can deliver a high-level expression of transgenes to almost all tissues and development stages in plants, which is particularly useful for the expression of herbicide tolerance, insect resistance and selectable marker genes [3]. For example, selectable marker gene systems are usually employed to avoid unnecessary and time-consuming screening for successful transgenic plants, which are more efficient at expressing selectable marker genes under the control of strong constitutive promoters [8, 9]; The *Bacillus thuringiensis* gene has been widely used to protect plants against insects in a dosage-dependent manner [10, 11]. Strong constitutive promoters facilitate the high expression of *Bacillus thuringiensis* gene, leading to an enhanced insect resistance [12], and the improtance of which has been verified by several studies in monocot or dicot plants, such as the cauliflower mosaic virus 35S (*CaMV35S*) promoter, the rice *Actin1* and cytochrome *c* gene promoter, and the maize *ubiquitin1* promoter [13–18]. Among these promoters, the *CaMV35S* promoter and maize *ubiquitin1* promoter are used most frequently to drive transgene expression in plants [19, 20]. However, they also have some drawbacks. The *CaMV35S* promoter drives high-level expression of transgenes in dicot plants, while the maize *ubiquitin1* promoter is more capable of driving gene expression in monocot plants [5]. Studies in rice have shown that the maize *ubiquitin1* promoter could drive efficient expression of transgenes in young roots and leaves, while the activity drastically decreased with aging [2]. The maize *ubiquitin1* promoter is inactive in some tissues in transgenic rice plants, such as anthers [21]. Due to the shortage of available promoters with the desired expression, the *CaMV35S* promoter is widely used to drive the expression of target genes and selectable marker genes in a single vector, such as pCAMBIA derivatives (http://www.cambia.org/daisy/cambia/materials/vectors.html), which greatly increases the occurrence of homologous-dependent gene silencing [7, 9, 22]. In addition, the *CaMV35S* promoter is derived from plant viruses. A number of limitations have been found during its application, such as its suppression in plant-parasitic nematode feeding sites [23, 24], public perception concerns with the use of virus-derived promoters [25], the ability of a cell to recognize the sequence as foreign and inactivate it [26], and its poor performance in monocots. Compared with virus- or bacteria-derived promoters, the promoters of plant origin may be safer and reduce gene silencing concerns [2]. Although there is little evidence to support the hypothetical risk of viral recombination events between the viral promoters and host’s DNA heavily utilized in plant genetic transformation [27–29]. The previous studies showed that a 19-bp palindromic sequence, including the TATA-box of *CaMV35S* promoter, acted as a recombination hotspot that was localized with the highly recombined region of *CaMV* RNA to promote viral recombination in dicots [30]. Therefore, the virus-derived promoters may have a greater potential risk than the plant endogenous promoters in plant genetic engineering. However, the majority of reported endogenous plant promoters are typically long and weak in directing gene expression, which restricts their application [1]. A shortage of available plant-derived promoters for high-level stable expression of foreign genes limits the development of genetically modified crops through transgenic technology.

Serine carboxypeptidase (SCP) and serine carboxypeptidase-like (SCPL) proteins comprise a large family of protein hydrolyzing enzymes that catalyze the hydrolysis of the C-terminal peptide bond in proteins or peptides [31, 32]. Several SCP/SCPL genes have been cloned from many plants, including rice [33–35], *Zea mays* [36], *Arabidopsis* [37, 38], *Pisum sativum* [39], wheat [40], oats [41] and tobacco [42], The studies have shown that SCP/SCPL genes are involved in a wide range of biological processes and are usually widely expressed in all tissues in higher plants. Among them, some members of *SCP/SCPL* are constitutively expressed, while some others are tissue-specifically expressed or induced by stress conditions. For example, Bienert et al. found that the serine carboxypeptidase genes *NtSCP1* and *NtSCP2* were widely expressed in the roots, stems, leaves and flowers of tobacco [42]; Liu et al. found that *OsBISCPL1*, a rice serine protein gene, was expressed in leaves, roots, stems and leaf sheaths [35]. The expression level of *OsBISCPL1* was relatively high in leaves and leaf sheaths and relatively weak in stems [35]. The previous studies showed that the serine carboxypeptidase-like 30 (*AtSCPL30*; At4g15100) is highly expressed in roots, leaves and flowers of *Arabidopsis thaliana* [43, 44]. However, *AtSCPL30* and its promoter region have not been characterized to date. Isolation and characterization of the *AtSCPL30* promoter will provide novel insights into understanding the transcriptional regulation of *AtSCPL30* and the promoter resources for crop genetic improvement by transgenic technology.

In this study, we describe the isolation and functional validation of the *AtSCPL30* promoter from *Arabidopsis thaliana* by deletion analysis in *Nicotiana benthamiana* transgenic plants. Our results showed that the *AtSCPL30* promoter (PD1) and its 5′ deletion fragments of different lengths (PD2-PD9) could confer strong and constitutive expression of β-glucuronidase (*GUS*) in almost all tissues and development stages in *Nicotiana benthamiana* transgenic plants. Among them, the abilities of PD2-PD7 to drive transgene expression were consistently more than twofold that of the *CaMV35S* promoter. The size of the PD7 fragment is only 456 bp in length. It will be a very useful tool for crop transgenic breeding, which provides a plant-derived alternative constitutive strong promoter.

## Methods

### Isolation of the *AtSCPL30* promoter from *Arabidopsis thaliana*

The 5′ flanking sequence of *AtSCPL30* (At4g15100) was retrieved from the TAIR database (http://www.arabidopsis.org/). The forward and reverse primers (named *P*_*AtSCPL30*FR_, Table 1**)** were designed according to the *AtSCPL30* sequence and its 5′ flanking region. The 2181-bp fragment (− 2093 ~ + 88 bp; the “A” of the translation start codon “ATG” of *AtSCPL30* was designated “+ 1”) was amplified from *Arabidopsis thaliana* genomic DNA with the *P*_*AtSCPL30*FR_ primers and confirmed by sequencing. Finally, a 2093-bp fragment upstream of the translation start codon of *AtSCPL30* was obtained by polymerase chain reaction (PCR) amplification using PD1FR primers (Table 1) and considered the full-length promoter (PD1).

**Table 1:** PCR primers used in the present study

Name	Forward (5′-3′)	Reverse (5′-3′)
*P*_*AtSCPL30FR*_	ctaatctcaatgtttccgcctttc	cacatgaaaccatttctactaatat
PD1FR	atcggatccctaatctcaatgtttccgc	att*ccatgg*ttgaggctaggttttagtag
PD2FR	atcggatccaagggaactacgcaaga	att*ccatgg*ttgaggctaggttttagtag
PD3FR	atcggatccctgctttcgatcatttc	att*ccatgg*ttgaggctaggttttagtag
PD4FR	atcggatccatggagttagagtttac	att*ccatgg*ttgaggctaggttttagtag
PD5FR	atcggatcctaagtcgatcaatccgc	att*ccatgg*ttgaggctaggttttagtag
PD6FR	atcggatccccgaatccacgaaac	att*ccatgg*ttgaggctaggttttagtag
PD7FR	atcggatccataggcaaccgtggact	att*ccatgg*ttgaggctaggttttagtag
PD8FR	atcggatccttatccctgtaaatcc	att*ccatgg*ttgaggctaggttttagtag
PD9FR	atcggatccgctaccaatttaccaca	att*ccatgg*ttgaggctaggttttagtag
*P*_*CaMV35SFR*_	aatggatccaagtctcaatagcccttt	tgagaattccgtattggctagagcagc
PD7-8FR	atcggatccataggcaaccgtggact	agc**ctgcag**attcgaatgaattcgtatat
PD8–9 FR	atcggatccttatccctgtaaatcct	aag**ctgcag**ggtagctttagtttattga
PD7-9FR	atcggatccataggcaaccgtggact	aag**ctgcag**ggtagctttagtttattga
HPTFR	cgtctgctgctccatacaa	tgtcctgcgggtaaatagc
*P*_*ZmUbi-1FR*_	gat*aagctt*ctgcagaagtaacaccaaacaatcg	agcggatccgcatgcctgcagtgcaactttatat

The underlined sites are the sites for the digestion of restriction enzymes *Bam*HI. The underlined italicized sites are the sites for the digestion of restriction enzymes *Nco*I. The sites in bold are the sitess for the digestion of restriction enzymes *Pst*I. The italicized site is the site for the digestion of restriction enzymes *Hin*dШ

### Bioinformatic analysis of the *AtSCPL30* promoter sequence

The potential *cis*-acting elements in the 2093-bp (− 2093 ~ − 1 bp) fragment of *AtSCPL30* promoter were analyzed with PLACE (http://www.dna.affrc.go.jp/PLACE/) [45] and PlantCARE (http://bioinformatics.psb.ugent.be/webtools/plantcare/html/) software [46], and were listed in Additional file 2: Figure S1 and Additional file 1: Table S1.

### Plasmid construction

**Fig. 1:**
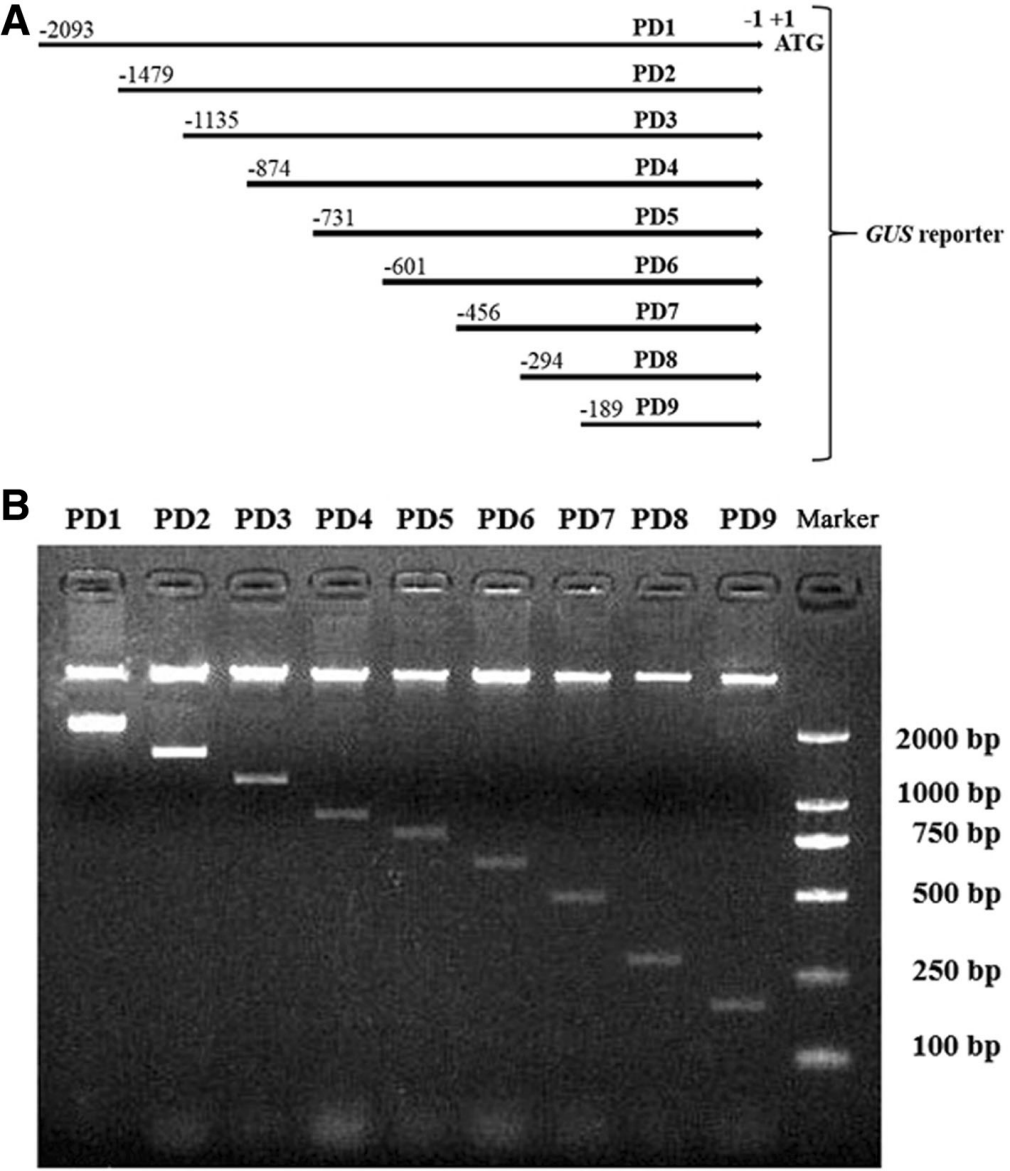
The constructs of the *AtSCPL30* promoter reporter plasmids and the picture of restriction digestion analysis. **a** The *AtSCPL30* promoter (PD1) and eight 5′ deleted fragments (PD2-PD9) were ligated upstream of the *GUS* reporter gene of the pCAMBIA1391Z vector. The numbers indicate the nucleotide position from the translational initiate code ATG (A as + 1). **b** The fused plasmids were confirmed by restriction digestion analysis with *Bam*HI/*Nco*I. Marker, DL2000

The *AtSCPL30* promoter (PD1, − 2093 bp to − 1 bp) and eight 5′ deleted fragments (PD2-PD9) in different lengths (− 1479 bp, − 1135 bp, − 874 bp, − 731 bp, − 601 bp, − 456 bp, − 294 bp, and − 189 bp to − 1 bp; Fig. 1a) were obtained by PCR amplification from the 2093-bp promoter region of *AtSCPL30* using the oligonucleotide primers listed in Table 1. For the construction of *AtSCPL30* promoter*::GUS* vectors, each PCR product was ligated into the pCAMBIA1391Z plasmid (Cambia, Australia) with *Bam*HI/*Nco*I restriction sites and confirmed by sequencing and restriction digestion (Fig. 1b). The resulting plasmids were used for the transformation of *Nicotiana benthamiana*. The pCAMBIA1304 (Cambia, Australia), a GUS expression vector driven by the *CaMV35S* promoter, was used as a positive control.

The mini35S plasmid was obtained as described by Hou et al. [6]. The 162-bp (− 456 ~ − 295 bp), 111-bp (− 294 ~ − 184 bp) and 273-bp (− 456 ~ − 184 bp) fragments from the *AtSCPL30* promoter sequence (the “A” of the translation start codon “ATG” was designated “+ 1”) were amplified by PCR using the primers PD7-8FR, PD8–9FR and PD7-9FR, respectively (Table 1), and then was confirmed by sequencing. Finally, the obtained 162-bp, 111-bp and 273-bp fragments were ligated into the mini35S plasmid with the *Bam*HI/*Pst*I restriction sites, respectively. The resulting plasmids were designated the PD7~ 8-mini35S, PD8~ 9-mini35S and PD7~ 9-mini35S and used for the transient assays in *Nicotiana benthamiana*.

### Culture and genetic transformation of *Nicotiana benthamiana*

The surface sterilization of *Nicotiana benthamiana* seeds, germination and culture of aseptic seedlings were performed as described by Hou et al. [6]. The leaves of 6-week-old seedlings were used for transformation.

The recombinant constructs PD1-PD9 and pCAMBIA1304 were transferred into *Agrobacterium tumefaciens* GV3101. The transformation of *Nicotiana benthamiana* were as described previously by Voelker et al. [47]. The transformed shoots were screened on MS medium containing 1.0 mg/L 6-benzylaminopurine, 0.1 mg/L indole-3-acetic acid, 300 mg/L cefotaxime and 15 mg/L hygromycin B. Regenerated shoots were rooted on MS medium supplemented with 200 mg/L cefotaxime and 15 mg/L hygromycin B. The regenerated seedlings were cultivated in nursery soil under 26–28 °C (day)/20–22 °C (night) with a cycle of 16-h light (230–260 μmol m^− 2^ s^− 1^). The T0 positive transformants were confirmed by PCR amplification (Additional file 3: Figure S2A) and GUS staining (Additional file 3: Figure S2B) as described by Hou et al. [6]. The T1-generation transgenic lines that displayed a 3:1 Mendelian segregation ratio by hygromycin B resistance screening were used for propagation (Additional file 4: Figure S3). Finally, the homozygous transgenic lines of the T3 generation with a single copy of the promoter*::GUS* insert from the *CaMV35S* and *AtSCPL30* promoter deletion vectors were selected for function analyses via segregation ratio analysis.

### NaCl, PEG and low-temperature stress treatments

PD1-PD9 and *CaMV35S* promoter transgenic and non-transgenic (WT) *Nicotiana benthamiana* seedlings were cultured as described by Hou et al. [6]. Finally, 60-day-old plants were subjected to NaCl, PEG and low-temperature stress treatments with either two fully expanded detached-leaves in vitro or intact plants.

The intact plants or the leaf discs 0.5 cm in diameter were treated with: (i) NaCl (0 and 200 mM) or polyethylene glycol (PEG) 6000 (0 and 18%, *w*/*v*) at 25 °C; or (ii) low temperature stress at 4 °C in the 1/2 MS liquid medium for 24 h, or 3, 6, 12, 24, 48, and 72 h, respectively. Leaf tissues were sampled for GUS staining immediately and frozen in liquid nitrogen for GUS fluorometric assays. At least three biological replicates for the independent experiment were performed.

### Histochemical and fluorometric GUS assays

Three independent transgenic lines and at least five individual plants from each plasmid were used for the *GUS* expression assay. GUS Histochemical staining and fluorometric assays were carried out as described by Jefferson et al. with minor modifications [48]. The tissues were incubated in GUS staining solution containing 50 mM sodium phosphate (pH 7.0), 0.5 mM potassium ferrocyanide, 0.5 mM potassium ferricyanide, 0.1% Triton X-100, 10 mM EDTA, and 1 mM X-Gluc (Sangon, Shanghai, China). Following vacuum infiltration, the samples were incubated at 37 °C for 3 h. After staining, the tissues were bleached with 70% ethanol and photographed (Sony DSC-F828 digital camera).

The tissues were homogenized in a 4 °C extraction buffer containing 50 mM sodium phosphate (pH 7.0), 10 mM DL-dithiothreitol, 0.1% sodium lauryl sarcosine, 10 mM EDTA and 0.1% Triton X-100 for GUS fluorometric assays. After centrifugation at 10000 *g* for 15 min at 4 °C, the activity of the supernatant was detected in an assay buffer containing 1 mM 4-methylumbelliferyl-b-glucuronide (4-MUG, Sigma, USA) at 37 °C. The reaction was terminated by the addition of 200 mM Na_2_CO_3_ to a final concentration of 180 mM. The fluorescence was quantified with a fluorescence spectrophotometer (HITACHI F-4600, Japan) at excitation and emission wavelengths of 365 nm and 455 nm, respectively. The protein concentration of the supernatant from each sample was determined as described by Bradford [49]. The GUS activity was normalized with five 4-MU standards (10 mM, 1 mM, 100 nM, 50 nM, and 10 nM) and calculated as nmol of 4-MU per mg protein per minute under control conditions.

### GUS transient assay in tobacco leaves

A GUS transient assay was performed using the leaves from sixty-day-old *Nicotiana benthamiana* plants as described by Yang et al. [50]. *A. tumefaciens* GV3101 harboring mini35S, PD7~ 8-mini35S, PD8~ 9-mini35S and PD7~ 9-mini35S plasmids were grown on YEP liquid medium containing 50 mg/L kanamycin and rifampin at 28 °C for 18 h. The harvest and resuspension of bacteria and infection of the leaves were performed as described by Hou et al. [6]. Finally, the leaf discs from twenty-three infiltrated plants were used for GUS fluorometric assays and GUS staining. The entire experiment was repeated three times.

### Transformation of maize calli

The sequence of the maize *ubiquitin1 promoter* was amplified from vector pTCK303 with the *P*_*ZmUbi-1FR*_ primers (Table 1) [51]. The amplified fragment was subsequently constructed into the vector pCAMBIA1391Z with *Hin*dШ/*Bam*HI restriction sites and confirmed by sequencing. The resulting plasmid pCAMBIA1391Z-Ubi-GUS was used as a positive control.

The constructs PD1, PD2, PD7 and pCAMBIA1391Z-Ubi-GUS were transferred into *Agrobacterium tumefaciens* strain LB4404 using a freeze-thaw method. The transgenic maize calli were obtained by *Agrobacterium* LB4404-mediated transformation of embryogenic type II calli from immature embryos of maize inbred line Qi319 as described by Quan et al. [52]. Transformed calli were selected on medium containing 15 mg/L hygromycin B for 2 weeks. Resistant calli were used for GUS histochemical staining.

### Data analysis

All GUS fluorometric assays were repeated at least three times. The results were expressed as the mean values ± SD (standard deviation). Student’s t test (*n* = 3, *P* < 0.05; Sigmaplot 12.0) at a 95% confidence level was used to test for statistical significance.

## Results

### Isolation of *AtSCPL30* promoter and sequence analysis

Based on the public sequence from TAIR (http://www.arabidopsis.org/), the 2093-bp promoter sequence of *AtSCPL30* (At4g15100) upstream of the start codon ATG was isolated from *Arabidopsis thaliana* genomic DNA. The *AtSCPL30* promoter sequence was analyzed through online programs PLACE and PlantCARE. The results showed that the 2093-bp promoter sequence contains twenty-six types of putative *cis*-acting elements (Additional file 2: Figure S1 and Additional file 1: Table S1), including CAAT-box and TATA-box core *cis*-acting elements. Moreover, some *cis*-acting elements that enable the tissue-specific, inducible, and enhanced or suppressed expression of *AtSCPL30* were identified, including five types of light-responsive elements (TCT-motif, GAG-motif, GATABOX, G-box and GT1CONSENSUS), six kinds of phytohormone-responsive elements (ABRE, NTBBF1ARROLB, WRKY71OS, TCA-element, SARE and CATATGGMSAUR), two kinds of elicitor-responsive elements (Box-W1 and EIRE), two kinds of elements required for tissue- or organ-specific expression (OSE1ROOTNODULE and TATCCAOSAMY), several elements involved in defense or stress (HSE, PREATPRODH, LTR, GT1GMSCAM4 and TC-rich repeats), five new signal element (POLASIG1), A negative *cis*-element conserved in plastid-related genes (S1FBOXSORPS1L21), five elements required for etiolation-induced expression of erd1 (ACGTATERD1) and 14 potential core sites required for the binding of Dof transcription factors that regulate the intensity of gene expression (DOFCOREZM). These potential elements may be related to the functional properties of the *AtSCPL30* promoter.

### Expression patterns and activities of the *AtSCPL30* promoter and its 5′ deletion segments in *Nicotiana benthamiana* transgenic plants under normal conditions

**Fig. 2:**
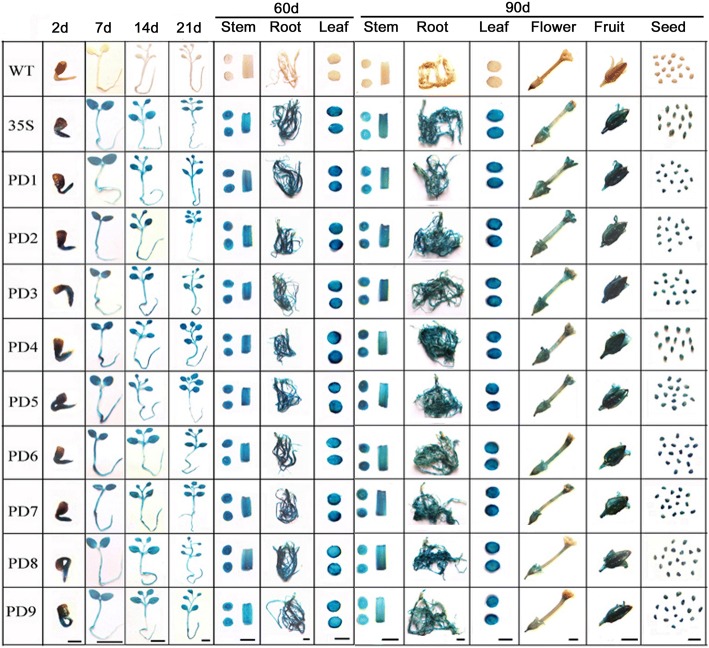
Histochemical GUS staining in tissues of PD1-PD9 and *CaMV35S* transgenic *Nicotiana benthamiana*. The 2-, 7-, 14-, and 21-day-old seedlings, leaves of 90-day-old plants, and roots, stems and leaves of 60-day-old plants were incubated in staining solution at 37 °C for 3 h. The roots, stems, flowers, fruits and seeds of 90-day-old plants were stained at 37 °C for 24 h. 35S, transgenic tobacco of the *CaMV35S* promoter. Scale bar of 2-day-old seedlings: 0.1 cm; the other scale bar: 0.5 cm

To profile the expression of *Nicotiana benthamiana* transgenic plants driven by PD1-PD9 and the *CaMV35S* promoter under normal conditions, the radicle of seeds germinated for 2 days; 7-, 14- and 21-day-old seedlings; the roots, stems, and leaves of 60- and 90-day-old plants; and the flowers, fruits and seeds from 90-day-old plants were subjected to GUS histochemical staining (Fig. 2). Our results showed that PD1-PD9 and the *CaMV35S* promoter were able to efficiently drive the expression in almost all the tissues of transgenic *Nicotiana benthamiana* at different developmental stages, except for the petals of PD8 and PD9.

Because of the original high GUS expression levels in various tissues of PD1-PD9 and *CaMV35S* promoter transgenic plants of *Nicotiana benthamiana*, it is difficult to see a significant difference in expression intensity just by GUS histochemical staining (Fig. 2). To clarify the difference in GUS expression levels between the series of mutants and *CaMV35S* promoter transgenic *Nicotiana benthamiana*, fluorometric GUS assays of the roots, stems and leaves from 60-day-old transgenic plants were carried out (Table 2). The results showed that the GUS activities in the roots, stems and leaves of PD1-PD9 and *CaMV3*5S promoter transgenic plants were higher than 80 nmol 4-MU/min•mg•protein under normal conditions, and there was no significant difference between the three tissues of each construct (Table 2). Among them, the GUS activity of PD1 (the full-length promoter) transgenic plants was approximately 1.7-fold that of the *CaMV35S* promoter; the GUS activities of PD2-PD7 were twofold higher than that of the *CaMV35S* promoter; GUS staining intensity of PD8 and PD9 was approximately 1.2-fold and 70% of *CaMV35S* promoter transgenic plants, respectively. Together, the above results indicated that the series of mutants of the *AtSCPL30* promoter were capable of directing GUS expression in almost all tissues at different developmental stages of transgenic *Nicotiana benthamiana* under normal conditions; specifically, the PD2-PD7 fragments were obviously higher than that of the *CaMV35S* promoter, showing a good potential for application in transgenic breeding.

**Table 2:** GUS fluorescent quantitative analysis of PD1-PD9 and *CaMV35S* promoter *Nicotiana benthamiana* transgenic plants under normal conditions

Item	GUS activities (nmol 4-MU/min•mg•protein)		
	Root	Stem	Leaf
*CaMV35S*	125.79 ± 9.81b	125.88 ± 2.63b	124.88 ± 2.61b
PD1	210.54 ± 4.74d	194.58 ± 7.85d	207.10 ± 9.23d
PD2	273.93 ± 7.79ef	280.61 ± 10.46ef	292.39 ± 2.01f
PD3	268.05 ± 4.7e	265.60 ± 3.74e	267.71 ± 9.61e
PD4	276.26 ± 10.38ef	279.41 ± 6.71ef	276.02 ± 7.38ef
PD5	263.96 ± 8.52e	274.32 ± 6.78ef	264.23 ± 2.18e
PD6	265.58 ± 9.47e	278.20 ± 3.21ef	260.38 ± 7.03e
PD7	270.56 ± 2.16ef	267.41 ± 3.55e	259.17 ± 5.14e
PD8	140.01 ± 9.80c	150.33 ± 4.47c	145.41 ± 9.53c
PD9	91.62 ± 9.78a	82.56 ± 6.13a	93.46 ± 6.23a

Values are means ± SD from 15 independent transgenic plants (5 individual plants/line, 3 lines for each construct). Different lowercase letters indicate significant differences at *P* < 0.05

### Characterization of the *AtSCPL30* promoter and its 5′ deletion segments in transgenic *Nicotiana benthamiana* plants under stress conditions

Under natural conditions, plants often suffer from adversities such as salinity, drought and low temperature. The sequence of the *AtSCPL30* promoter contained some potential elements involved in low-temperature responsiveness, salt-induced gene expression and osmotic stress. Further work was carried out to investigate whether the *AtSCPL30* promoter and its 5′ deletion segments were active under various stress conditions. The abilities of PD1-PD9 to drive gene expression were detected in the leaves of *Nicotiana benthamiana* transgenic plants under low-temperature, salt- or osmotic-stress treatments. Positive (*CaMV35S* promoter transgenic plants) and negative (non-transformed wild-type plants) control materials were also treated in parallel.

The detached leaf discs from sixty-day-old transgenic plants were treated under 200 mM NaCl (Additional file 5: Figure S4), 18% PEG 6000 (*w*/*v*, Additional file 6: Figure S5) or low temperature (4 °C, Additional file 7: Figure S6) for 3, 6, 12, 24, 48, and 72 h, respectively. The intensity of GUS staining in the leaf discs of PD1-PD9 and *CaMV35S* promoter transgenic plants were no obvious differences between the control and stress-treated groups. However, it is worth noting that PD1-PD9 was still able to confer high levels of GUS expression during the NaCl, PEG and 4 °C treatments.

**Fig. 3:**
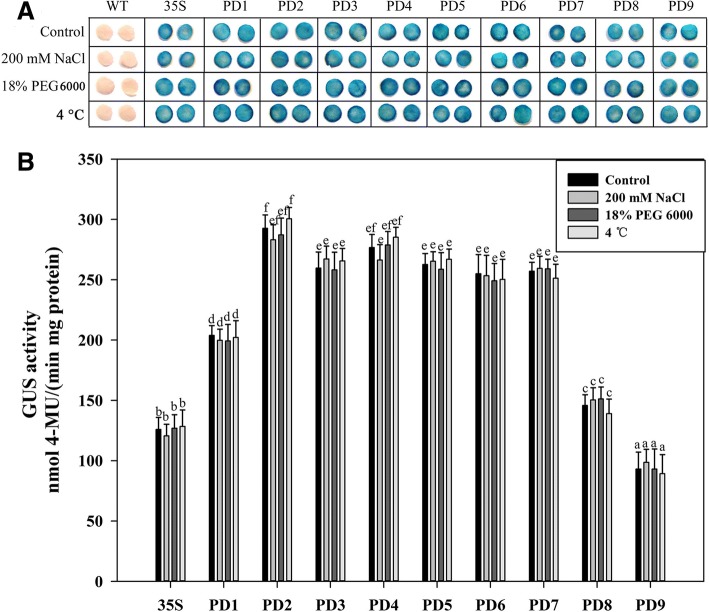
GUS staining and fluorescent quantitative analysis of PD1-PD9 *Nicotiana benthamiana* transgenic plants under normal and stress conditions. For NaCl and PEG treatments of whole plants, 60-day-old transgenic plants were immersed in a liquid 1/2 MS medium supplemented with either 200 mM NaCl or 18% (*w*/*v*) PEG 6000 at 25 °C for 24 h. For 4 °C treatment of whole plants, 60-day-old plants were immersed in a liquid 1/2 MS medium at 4 °C for 24 h. The plants grown in liquid 1/2 MS medium at 25 °C for 24 h were treated as a control. **a** GUS histochemical staining. The leaves of PD1–PD9 and *CaMV35S* promoter transgenic plants were stained for 3 h. Samples were then observed and photographed after decolorization. **b** GUS activity assays of leaves. Values represent the means ± SD from 15 independent transgenic plants (5 individual plants/line, 3 lines for each construct). Different lowercase letters above the bars indicate significant differences at *P* < 0.05

To further confirm the above results, the stress treatments involving whole plants of sixty-day-old transgenic and non-transformed wild-type *Nicotiana benthamiana* were also carried out. Based on the experiment results of detached-leaves, the *Nicotiana benthamiana* plants were chosen to treat 24 h under 200 mM NaCl, 18% PEG 6000 (*w*/*v*) or 4 °C condition. GUS staining and fluorometric assay showed that the promoter activities of PD1-PD9 were indeed not induced by salt stress, osmotic stress and 4 °C treatments, whereas the *AtSCPL30* promoter and its 5′ deletion segments were capable of maintaining a high-level expression of the GUS gene under severe stress conditions (Fig. 3). Thereby, PD1-PD9 can efficiently drive the expression of foreign genes both before and after stresses. Notably, the PD2-PD7 fragments exhibited higher levels of promoter activity in *Nicotiana benthamiana* transgenic plants before and after the stress treatments compared to the full-length promoter (PD1) and PD8 and PD9 fragments, which were twofold higher than those of the *CaMV35S* promoter (Fig. 3b). Among them, PD7 was only 456 bp in length, and its promoter activity was comparable to that of PD2-PD6 (Fig. 3b). Therefore, PD7 may be the core functional segment that confers strong constitutive expression of the *AtSCPL30* promoter. It could be of great use to drive transgene expression based on its expression patterns, promoter activity, stability and small size.

### The 162-bp, 111-bp and 273-bp fragments all contribute significantly to gene expression

**Fig. 4:**
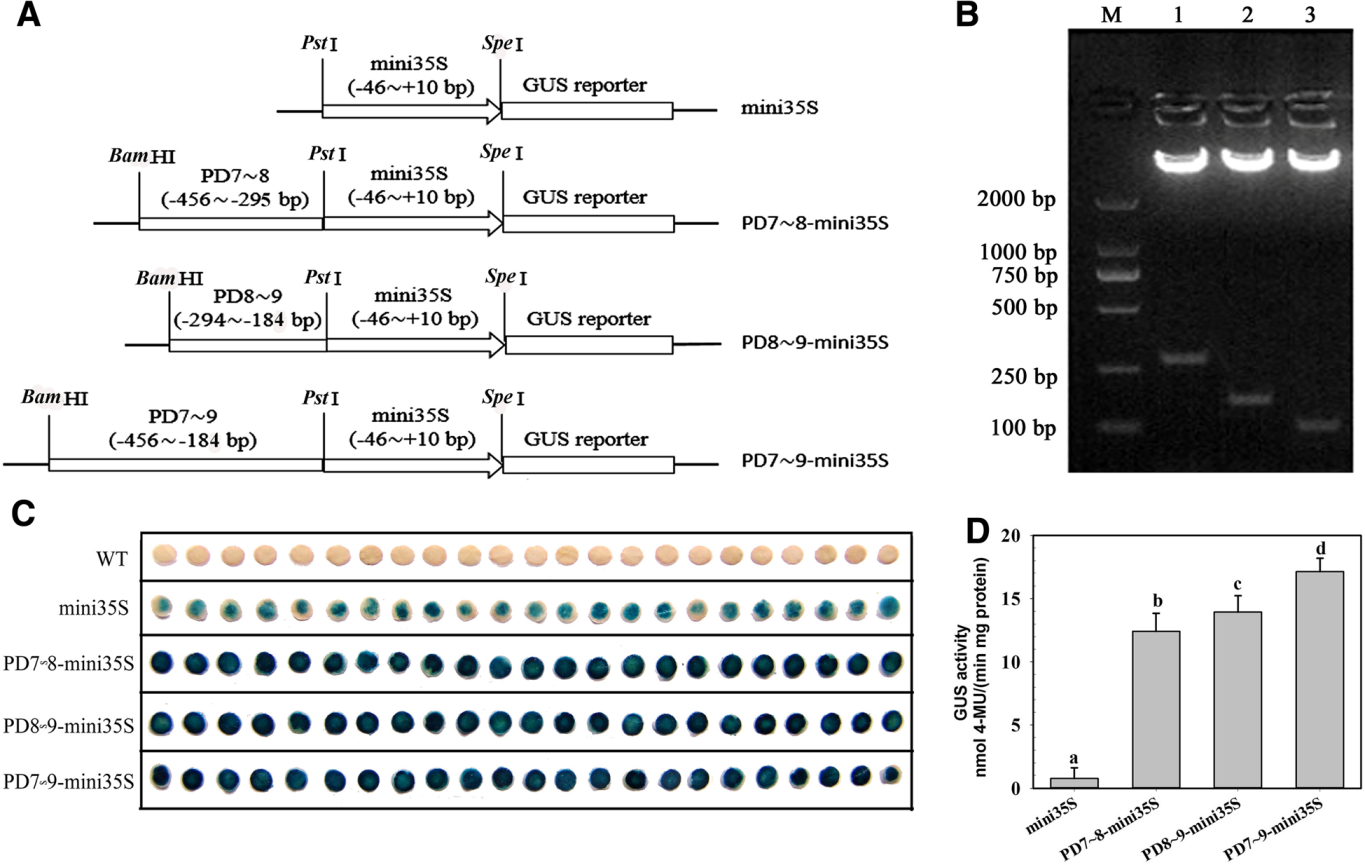
GUS transient assays in the leaves of *Nicotiana benthamiana* plants. **a** The plasmids used in the transient assay. Mini35S represents the truncated *CaMV35S* promoter (− 46 ~ + 10 bp). The test constructs consisted of PD7~ 8-mini35S, PD8~ 9-mini35S and PD7~ 9-mini35S, in which the 162-bp (− 456 ~ − 295 bp), 111-bp (− 294 ~ − 184 bp) and 273-bp (− 456 ~ − 184 bp) fragments identified in the *AtSCPL30* promoter were fused to the mini35S promoter to drive GUS expression. **b** The fused plasmids were confirmed by restriction digestion analysis with *Bam*HI/*Pst*I. M, Molecular marker DL2000; 1, PD7~ 9-mini35S; 2, PD7~ 8-mini35S; 3, PD8~ 9-mini35S. **c** Histochemical GUS staining resulting from non-transformed leaves (WT) and transiently transformed leaves with constructs mini35S, PD7~ 8-mini35S, PD8~ 9-mini35S and PD7~ 9-mini35S under normal conditions. **d** GUS activity in the transiently transformed tobacco leaves with constructs mini35S, PD7~ 8-mini35S, PD8~ 9-mini35S and PD7~ 9-mini35S under normal conditions. The results are the mean ± SD from three experiments (*n* = 23). Different lowercase letters above the bars indicate significant differences at *P* < 0.05

The abilities of PD8 and PD9 to drive gene expression were significantly lower than that of PD2-PD7 in both before and after stresses (Table 2 and Fig. 3b). The promoter activity of PD8 was approximately 60% of that of PD7. The ability of PD9 to initiate gene expression was approximately 65% of the PD8 and 35% of the PD7. The results suggested that the 162-bp fragment (− 456 ~ − 295 bp), the 111-bp fragment (− 294 ~ − 184 bp), and the 273-bp sequence (− 456 ~ − 184 bp) may be able to promote gene expression. The 162-, 111- and 273-bp fragments were fused with mini35S (− 46 ~ + 10 bp) to drive GUS expression (Fig. 4a and b). The GUS transient assay in leaves of *Nicotiana benthamiana* plants showed that the 162-, 111- and 273-bp fragments increased the ability of mini35S to drive GUS expression by 16-, 18- and 22-fold, respectively (Fig. 4c and d). These segments will provide a sequence source for the artificial modification of promoters in the future.

### Detection of promoter activities of PD1, PD2 and PD7 in maize calli

**Fig. 5:**
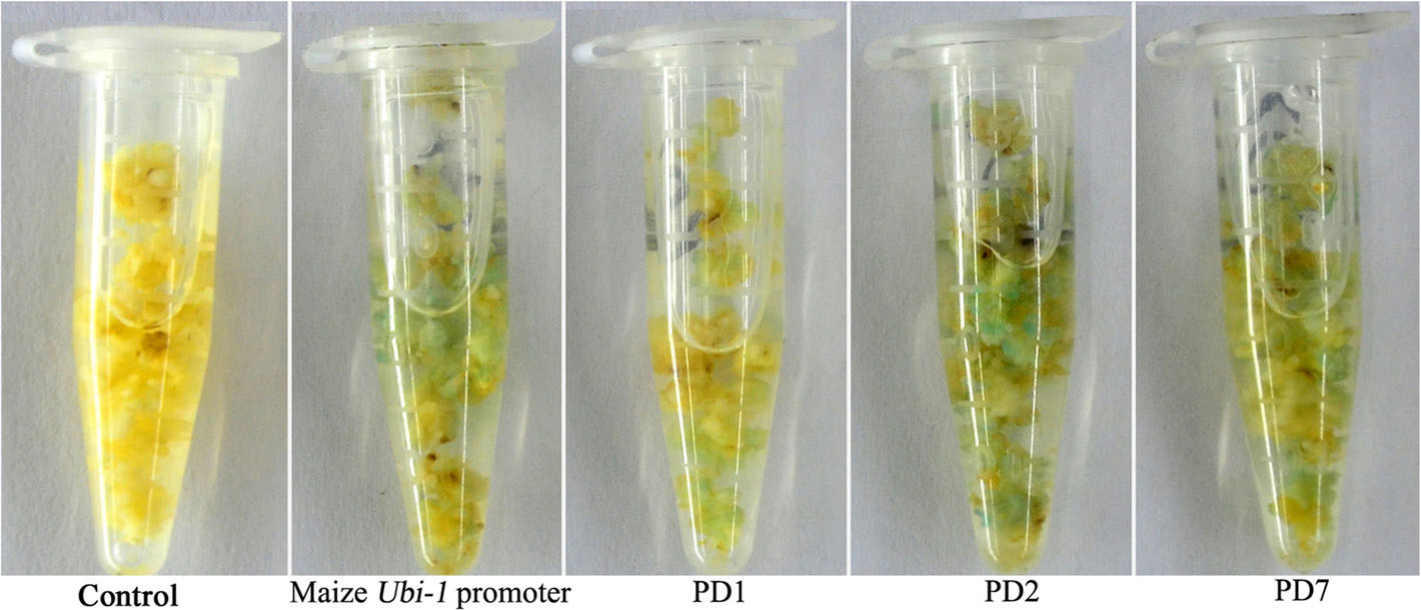
GUS histochemical staining of transformed maize calli. Resistant calli were incubated in staining solution at 37 °C for 24 h. Finally, the samples were observed and photographed after decolorization

To preliminarily evaluate its application in the genetic transformation of monocots, the full-length promoter PD1, the fragments of PD2 and PD7, and the maize *ubiquitin1* promoter (positive control) were selected to construct into the vector pCAMBIA1391Z driving the expression of *GUS* and introduced into maize calli. GUS histochemical staining showed that the PD2 and PD7 fragments seem to have a higher ability to drive gene expression than the maize *ubiquitin1* promoter in maize calli (Fig. 5).

## Discussion

Promoters play important roles in initiating gene transcription and regulating the expression of transgenes. Therefore, a good knowledge of promoter transcriptional activities and expression patterns is necessary for the successful application of transgenic breeding and gene function studies [5, 53]. In this study, the *AtSCPL30* promoter from *Arabidopsis thaliana* was isolated and characterized by deletion analysis in *Nicotiana benthamiana* transgenic plants. We found that the *AtSCPL30* promoter (PD1) and its 5′ deletion fragments (PD2-PD9) were ubiquitously expressed in different tissues of *Nicotiana benthamiana* and retained their activities during high salt, osmotic stress and low-temperature treatment. Finally, a 456 bp (PD7) core functional segment of the *AtSCPL30* promoter was identified. The promoter activity of PD7 was higher than that of PD1, PD8 and PD9 and comparable to that of PD2-PD6, which is more than twofold higher than the *CaMV35S* promoter in different tissues of *Nicotiana benthamiana* under normal and stress conditions (Fig. 3b). Unlike the *CaMV35S* promoter from plant viruses, PD7 is a small plant endogenous constitutive promoter. Thus, it has the advantage of biosafety and reduces the probability of transgene silencing and the vector size, which is beneficial for genetic transformation [2]. Moreover, the stress adaptability of crops is quite complex. Single transgene introduction may not be sufficient to improve crop adaptability under natural conditions. However, multiple transgenes are introduced into plants and their expression is regulated using the same promoter in the construct, such as the *CaMV35S* promoter, which will greatly increase the occurrence of homologous-dependent gene silencing [3, 54]. Therefore, isolation and functional validation of more alternative constitutive promoters to the *CaMV35S* promoter is highly desirable. PD7 is an alternative constitutive strong promoter to the *CaMV35S* promoter and will be a very useful tool for transgenic breeding.

**Fig. 6:**
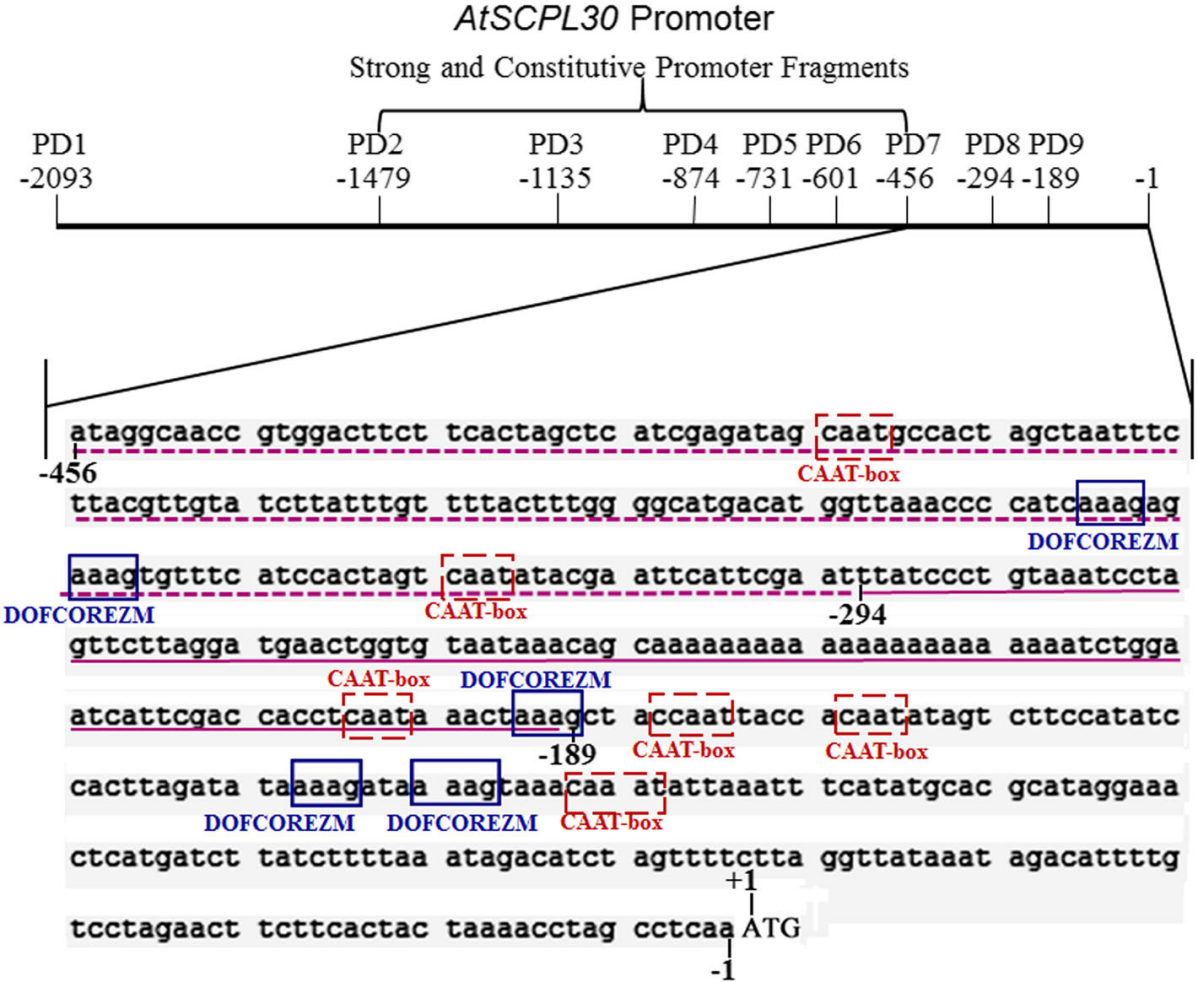
Diagrams of PD7 promoter fragment and the 163- and 106-bp fragments for enhanced gene expression. Potential *cis*-acting elements associated with increased gene expression in the PD7 sequence predicted by PlantCARE and PLACE are shown on the border. CAAT box, common *cis*-acting element in promoter and enhancer regions; DOFCOREZM, core site required for binding of Dof proteins in maize. Dof1 and Dof2 transcription factors are associated with the expression of multiple genes involved in carbon metabolism in maize

Promoters usually regulate the intensity of gene expression through the interaction of some specific *cis*-acting elements on the sequence with its interacting proteins, such as transcription factors. PD7 has very high promoter activity in *Nicotiana benthamiana* transgenic plants, while the deletion of the 163-bp fragment (− 456 ~ − 294 bp) between PD7 and PD8 and the 106-bp fragment (− 294 ~ − 189 bp) between PD8 and PD9 resulted in a significant decrease in GUS activity (Fig. 3b). The results showed that these fragments mediated transcriptional enhancement and contributed to the strong promoter activities of PD2-PD7 fragments in *Nicotiana benthamiana* transgenic plants. The GUS transient assay in the leaves of 60-day-old *Nicotiana benthamiana* plants also showed that they have a strong ability to enhance gene expression (Fig. 4c and d). Bioinformatic analysis determined that the PD7 sequence, including the 163-bp and 106-bp regions, contains several elements (such as six CAAT-box and five DOFCOREZM domains) related to enhancing gene expression (Fig. 6). The CAAT-box is a common *cis*-acting element in promoter and enhancer regions that typically exhibits a putative effect in enhancing gene expression. DOFCOREZM is one of the binding site of Dof proteins [55, 56]. Dof1 and Dof2 have been find to regulate the expression of multiple genes involved in carbon metabolism in maize, such as Dof1 can bind to the promoter of both cytosolic orthophosphate kinase (CyPPDK) and a non-photosynthetic PEPC gene to enhance their expression [57]. It has been confirmed that the Dof factor binding sites in subdomain B4 of the *CaMV35S* promoter are important and contribute to its promoter activity [20]. Indeed, much of the fine-tuning of gene expression is controlled by sequence-targeted enhancer-binding proteins or transcription factors, which bind to *cis*-elements within the promoter [2]. Based on the above results, we speculated that the CAAT-box and DOFCOREZM elements may be important factors contributing to the high promoter activity of PD7 and enhancer activities of the 162-bp and 111-bp fragments. Moreover, the deletion of the 615-bp (− 2093 ~ − 1479) sequence between PD1 and PD2 resulted in a significant increase of GUS activity (PD2-PD7) in *Nicotiana benthamiana* transgenic plants. The results showed that the 615-bp sequence mediates the transcriptional repression and contributes to relatively weak promoter activity of PD1 compared to PD2-PD7. We think that the 615-bp fragment may contain *cis*-acting elements that inhibit gene expression. The deletion of the 163-bp fragment (− 456 ~ − 294 bp) between PD7 and PD8 causes PD8 and PD9 to not effectively drive gene expression in petals (Fig. 2). The results suggested that the 163-bp sequence contains the elements required for petal tissue-specific expression and contributes to promoter activity of PD1-PD7 fragments in petals of *Nicotiana benthamiana*.

In addition, the expression pattern and transcriptional activity of promoters may have obvious differences in dicot and monocot. The *CaMV35S* promoter is a widely used constitutive promoter conferring the strong expression of transgenes in dicots, while the ubiquitin promoter usually drives gene expression more efficiently in monocots than the *CaMV35S* promoter. For example, the maize *ubiquitin1* promoter conferred gene expression up to or over tenfold higher than the *CaMV35S* promoter in rice, maize, wheat and barley, but its ability to drive gene expression in tobacco protoplasts was less than one-tenth of that of the *CaMV35S* promoter [4, 58, 59]. However, there are also some promoters that displayed high promoter activity in both monocots and dicots. For example, the small *AtCTTP* promoter (0.3 kb) displayed high transcriptional activities in *creeping bentgrass* (monocot) and *Arabidopsis* (dicot) [3]. The results suggested that the small *AtCTTP* promoter could be used in both monocot and dicot plant transgenic breeding. In this study, we identified PD2-PD7 fragments from the *AtSCPL30* promoter that conferred strong and constitutive gene expression in *Nicotiana benthamiana* (dicot), implying that these fragments would be very useful tools for transgenic breeding in dicot. Using PD2 and PD7 fragments to drive GUS expression in maize calli, the results showed that they appeared to have higher promoter activities than maize *ubiquitin1* promoter (Fig. 5). Further characterization of the expression patterns and transcriptional activities of PD2 and PD7 fragments in maize and evaluation of their application prospects in the transgenic breeding of monocot crops will also be meaningful in the future.

## Conclusions

In this study, we isolated and characterized the promoter from the *Arabidopsis* serine carboxypeptidase-like gene (*AtSCPL30*) in *Nicotiana benthamiana* transgenic plants. By analyzing the *AtSCPL30* promoter (PD1) and its 5′ deleted fragments (PD2-PD9) under normal and stress conditions, we found that PD2-PD7 fragments could confer strong and constitutive expression of foreign genes and that their transcriptional activities were consistently over twofold higher than the well-used *CaMV35S* promoter in *Nicotiana benthamiana* transgenic plants (Fig. 3b). Among them, PD7 was only 456 bp in length, and its transcriptional activity was comparable to that of PD2-PD6. The PD7 sequence has also been shown to have very high transcriptional activity in maize calli (Fig. 5). Based on its expression patterns, promoter activity, small size and stability, the constitutive strong promoter (PD7) of plant origin is very useful for avoiding the repetitive usage of the virus-derived *CaMV35S* promoter and reducing the vector size and the probability of transgene silencing; thus, this promoter is beneficial for the successful application of transgenic technology.

Furthermore, the 162-bp (− 456 ~ − 295 bp), 111-bp (− 294 ~ − 184 bp) and 273-bp (− 456 ~ − 184 bp) fragments from the *AtSCPL30* promoter were identified as the regions for enhancing gene expression by 5′ deletion mutation analysis, with a GUS transient assay in transformed leaves of *Nicotiana benthamiana* plants revealing that they do have very high enhancer activity. These sequences will provide important *cis*-regulatory elements for synthetic promoter design based on their enhancer activities.

## Additional files

Additional file 1: **Table S1.** Potential *cis*-acting elements in the *AtSCPL30* promoter sequence using the PLACE and PlantCARE databases. (DOCX 17 kb)

Additional file 2: **Figure S1.** Nucleotide sequence of the *AtSCPL30* promoter. The “A” of the translation initiation code “ATG” of the *AtSCPL30* was designated as “+ 1”. Potential *cis*-acting elements underlined, different color or shown in the border. See Table S1 for descriptions of the elements. The arrow above the sequence indicates the start point of PD1 and different deletion fragments (PD2-PD9). (JPG 3013 kb)

Additional file 3: **Figure S2.** The PCR analysis and histochemical GUS staining of *Nicotiana benthamiana* transgenic plants. (A) Genomic PCR analysis of transformed plants using primers HPTFR (Table 1) designed for the hygromycin gene. Marker, DL2000 plus; +, the PCR result of plasmid pCAMBIA1391Z; WT, non-transformed control; 1–12, transformed T0 plants. (b) Histochemical GUS staining of transgenic plants. WT, non-transformed plants; 35S, transgenic plants of *CaMV35S* promoter; PD1-PD9, transgenic plants of PD1 and eight truncated promoter fragments (PD2-PD9). (JPG 524 kb)

Additional file 4: **Figure S3.** The analysis of genetic segregation ratio in *Nicotiana benthamiana* transgenic plants by hygromycin resistance screening. (JPG 2214 kb)

Additional file 5: **Figure S4.** GUS staining of detached leaves of *Nicotiana benthamiana* transgenic plants under normal and salt-stress conditions. Ninety leaf discs (diameter 0.5 cm) from fifteen 60-day-old individual plants (5 individual plants/ line, 3 lines for each construct) of PD1-PD9 and *CaMV35S* transgenic plants were incubated in liquid 1/2 MS medium supplemented with 200 mM NaCl at 25 °C for 3, 6, 12, 24, 48, and 72 h; leaf discs floated in liquid 1/2 MS medium were used as control. The leaf discs of PD1-PD9 and *CaMV35S* transgenic plants were then incubated in staining solution at 37 °C for 3 h. Finally, the samples were observed and photographed after decolorization. (JPG 3572 kb)

Additional file 6: **Figure S5.** GUS staining of detached leaves of *Nicotiana benthamiana* transgenic plants under normal and PEG treatment conditions. Ninety leaf discs (diameter 0.5 cm) from fifteen 60-day-old individual plants (5 individual plants/line, 3 lines for each construct) of PD1-PD9 and *CaMV35S* transgenic plants were incubated in liquid 1/2 MS medium supplemented with 18% PEG6000 (*w*/*v*) at 25 °C for 3, 6, 12, 24, 48, and 72 h; leaf discs floated in liquid 1/2 MS medium were used as control. The leaf discs of PD1-PD9 and *CaMV35S* transgenic plants were then incubated in staining solution at 37 °C for 3 h. Finally, the samples were observed and photographed after decolorization. (JPG 2139 kb)

Additional file 7: **Figure S6.** GUS staining of detached leaves of *Nicotiana benthamiana* transgenic plants under normal and low-temperature conditions. Ninety leaf discs (diameter 0.5 cm) from fifteen 60-day-old individual plants (5 individual plants/line, 3 lines for each construct) of PD1-PD9 and *CaMV35S* transgenic plants were incubated in liquid 1/2 MS medium at 4 °C for 3, 6, 12, 24, 48, and 72 h; leaf discs floated in liquid 1/2 MS medium at 25 °C were used as control. The leaf discs of PD1-PD9 and *CaMV35S* transgenic plants were then incubated in staining solution at 37 °C for 3 h. Finally, the samples were observed and photographed after decolorization. (JPG 2225 kb)

## Abbreviations

*AtSCPL30*: *Arabidopsis thaliana* serine carboxypeptidase-like 30
*CaMV35S*: Cauliflower mosaic virus 35S
GUS: β-glucuronidase
PEG: polyethylene glycol
SCP: Serine carboxypeptidase
SCPL: Serine carboxypeptidase-like
