# Supplementary material for: Characterization of a strong and constitutive promoter from the Arabidopsis serine carboxypeptidase-like gene AtSCPL30 as a potential tool for crop transgenic breeding
Source: BMC Biotechnol. 2018 Sep 21;18:59. doi: 10.1186/s12896-018-0470-x (PMC6151023; doi:10.1186/s12896-018-0470-x)
Supplement: Supplementary file 1 — Table S1. Potential cis-acting elements in the AtSCPL30 promoter sequence using the PLACE and PlantCARE databases. (DOCX 17 kb) [file 12896_2018_470_MOESM1_ESM.docx]

**Table S1** Potential *cis*-acting elements in the *AtSCPL30* promoter sequence using the PLACE and PlantCARE databases.

| Cis-elements | Description | Position from ATG | No. |
| --- | --- | --- | --- |
| HSE | *Cis*-acting element involved in heat stress responsiveness | -828 | 1 |
| TCT-motif | Part of a light responsive element | -708, -397 | 2 |
| GAG-motif | Part of a light responsive element | -1988 | 1 |
| POLASIG1 | A new signal element | -1776, -831, -680, -255, -200 | 5 |
| PREATPRODH | Pro- or hypoosmolarity-responsive element | -777, -97 | 2 |
| LTR | *Cis*-acting element involved in low-temperature responsiveness | -1619, -478 | 2 |
| ABRE | *Cis*-acting element involved in the abscisic acid responsiveness | -528 | 1 |
| GT1GMSCAM4 | Plays a role in pathogen-and salt-induced SCaM-4 gene expression | -921, -684 | 2 |
| NTBBF1ARROLB | Required for tissue-specific expression and auxin induction | -859 | 1 |
| GATABOX | *Cis*-acting regulatory element involved in light responsiveness | -1993, -1926, -768, -733, -421, -150, -141 | 7 |
| S1FBOXSORPS1L21 | A negative *cis*-element conserved in plastid-related genes | -1666 | 1 |
| OSE1ROOTNODULE | The consensus sequence motifs of organ-specific elements | -144 | 1 |
| ACGTATERD1 | Required for etiolation-induced expression of erd1 | -2024, -1838, -1379, -1057, -394 | 5 |
| WRKY71OS | A transcriptional repressor of the gibberellin signaling pathway | -1609, -362 | 2 |
| G-box | *Cis*-acting regulatory element involved in light responsiveness | -2023 | 1 |
| TCA-element | *Cis*-acting element involved in salicylic acid responsive-ness | -1709 | 1 |
| SARE | *Cis*-acting element involved in salicylic acid responsiveness | -1532 | 1 |
| Box-W1 | Fungal elicitor responsive element | -1386 | 1 |
| DOFCOREZM | Core site required for binding of Dof proteins in maize. Dof1 and dof2 transcription factors are associated with expression of multiple genes involved in carbon metabolism in maize. | -1990, -1877, -1585, -1424, -1310, -1209, -969, -697, -617, -342, -336, -192, -144, -137 | 14 |
| CATATGGMSAUR | Involved in auxin responsiveness | -1920, -653, -115 | 3 |
| TATCCAOSAMY | Required for tissue-specific expression | -1867, -1818, -1642, -160 | 4 |
| EIRE | Elicitor-responsive element | -212 | 1 |
| GT1CONSENSUS | *Cis*-acting regulatory element involved in light responsiveness | -356 | 1 |
| TC-rich repeats | *Cis*-acting element involved in defense and stress | -65 | 1 |
| CAAT-box | Common cis-acting element in promoter and enhancer regions | -2086, -1638, -1434, -1326, -1259, -1252, -1030, -816, -722, -693, -416, -316, -201, -185, -175, -129 | 16 |
| TATA-box | Core promoter element around -30 of transcription start | -1869, -1803, -1669, -1394, -1234, -1187, -1012, -1003, -953, -925, -795, -377, -146, -82, -53 | 15 |

The numbers indicate the nucleotide position from the translational initiate site, ATG (A as +1).
