# Supplementary figures and images for: Characterization of a strong and constitutive promoter from the Arabidopsis serine carboxypeptidase-like gene AtSCPL30 as a potential tool for crop transgenic breeding
Source: BMC Biotechnol. 2018 Sep 21;18:59. doi: 10.1186/s12896-018-0470-x (PMC6151023; doi:10.1186/s12896-018-0470-x)

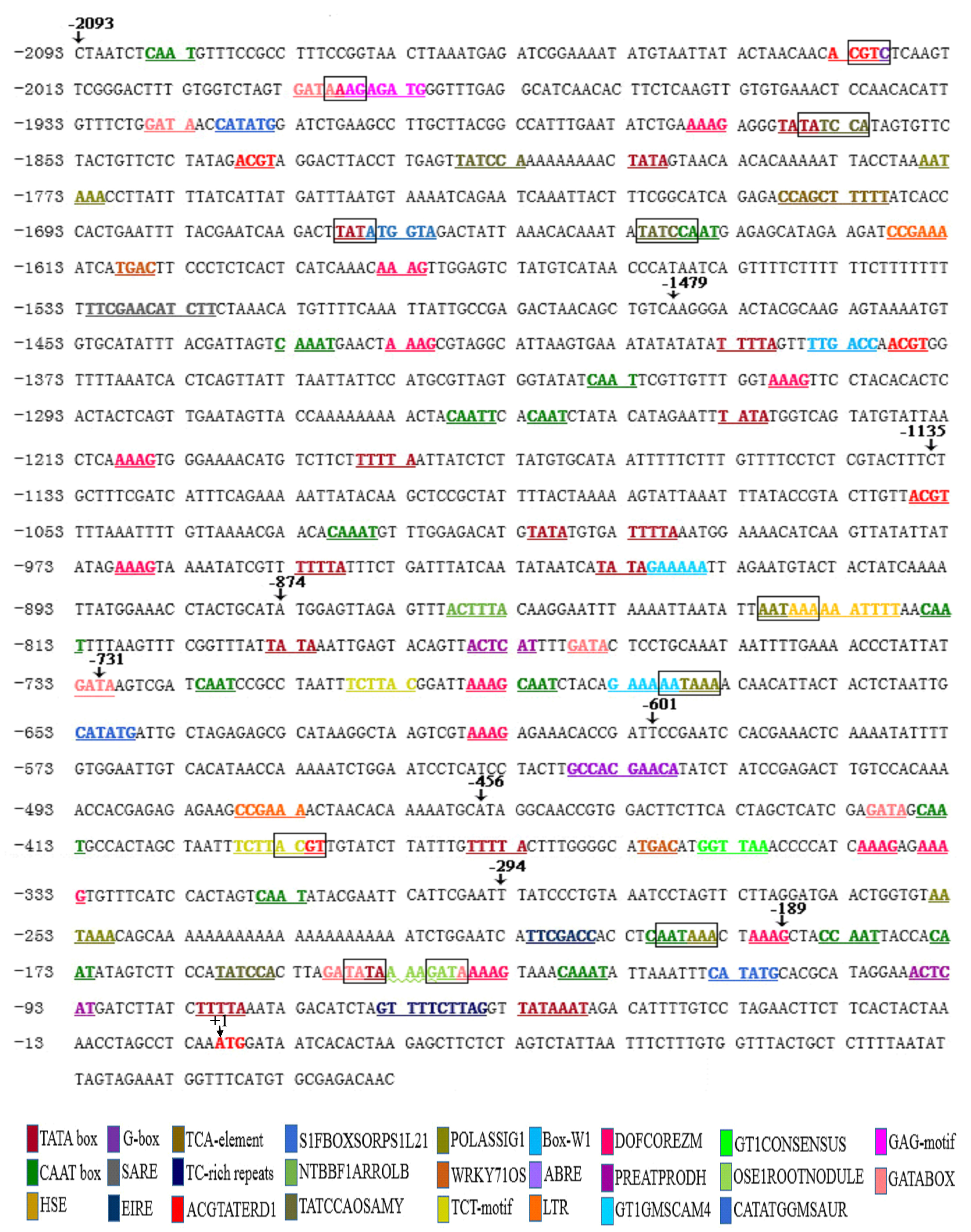

Supplement: Supplementary file 2 — Figure S1. Nucleotide sequence of the AtSCPL30 promoter. The “A” of the translation initiation code “ATG” of the AtSCPL30 was designated as “+ 1”. Potential cis-acting elements underlined, different color or shown in the border. See Table S1 for descriptions of the elements. The arrow above the sequence indicates the start point of PD1 and different deletion fragments (PD2-PD9). (JPG 3013 kb) [file 12896_2018_470_MOESM2_ESM.jpg]

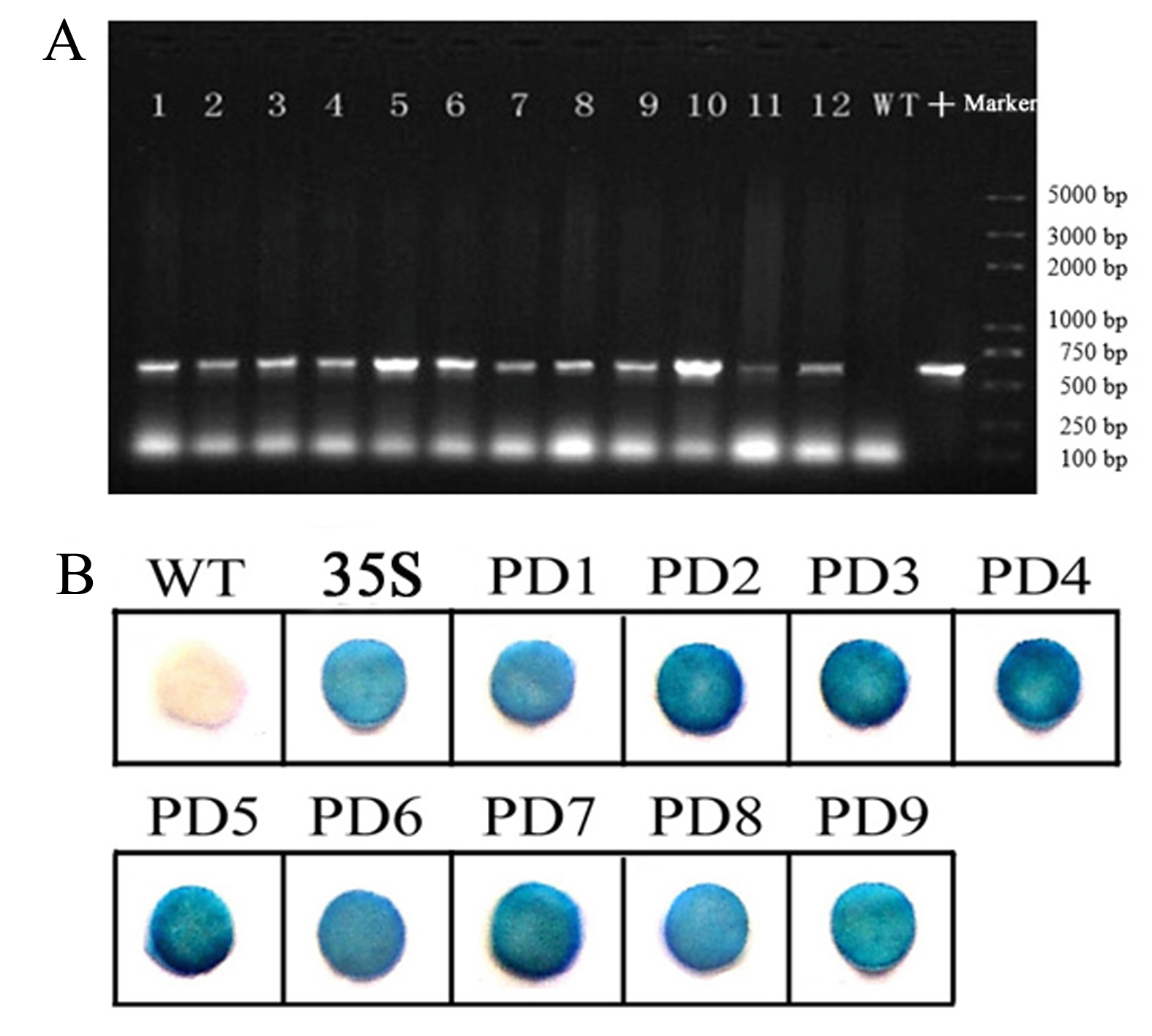

Supplement: Supplementary file 3 — Figure S2. The PCR analysis and histochemical GUS staining of Nicotiana benthamiana transgenic plants. (A) Genomic PCR analysis of transformed plants using primers HPTFR (Table 1) designed for the hygromycin gene. Marker, DL2000 plus; +, the PCR result of plasmid pCAMBIA1391Z; WT, non-transformed control; 1–12, transformed T0 plants. (b) Histochemical GUS staining of transgenic plants. WT, non-transformed plants; 35S, transgenic plants of CaMV35S promoter; PD1-PD9, transgenic plants of PD1 and eight truncated promoter fragments (PD2-PD9). (JPG 524 kb) [file 12896_2018_470_MOESM3_ESM.jpg]

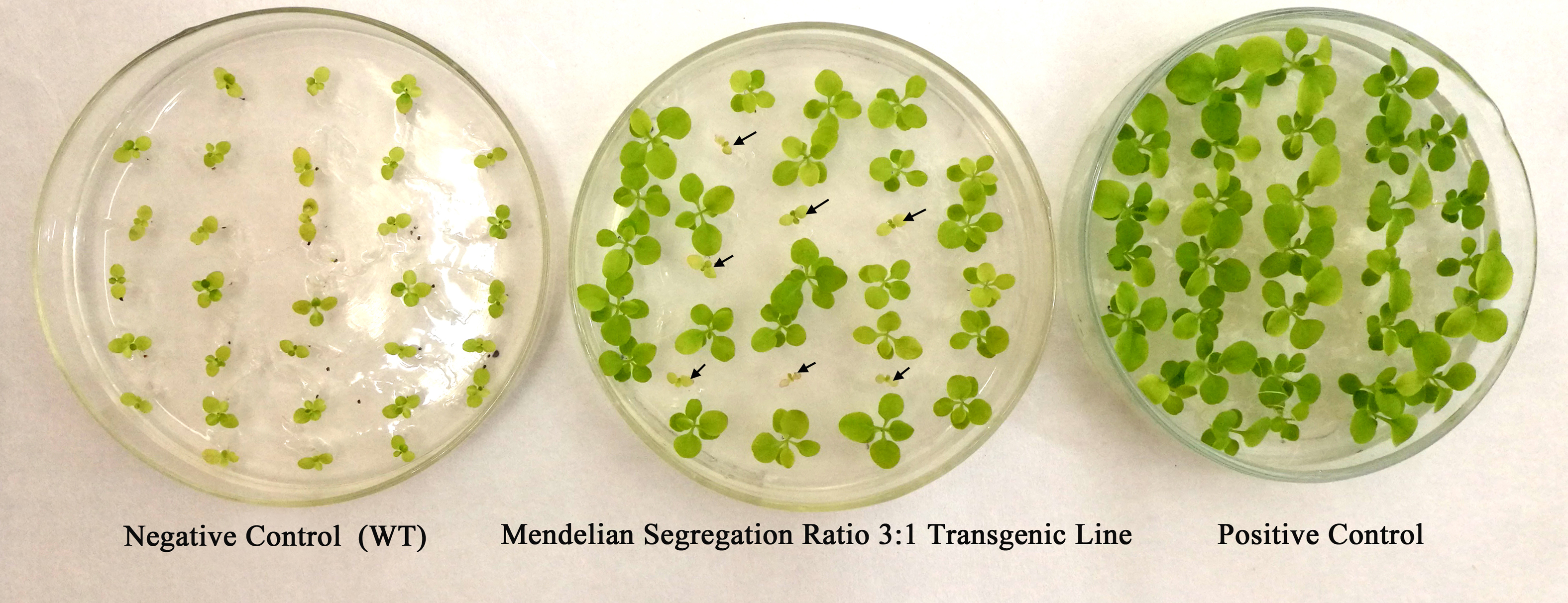

Supplement: Supplementary file 4 — Figure S3. The analysis of genetic segregation ratio in Nicotiana benthamiana transgenic plants by hygromycin resistance screening. (JPG 2214 kb) [file 12896_2018_470_MOESM4_ESM.jpg]

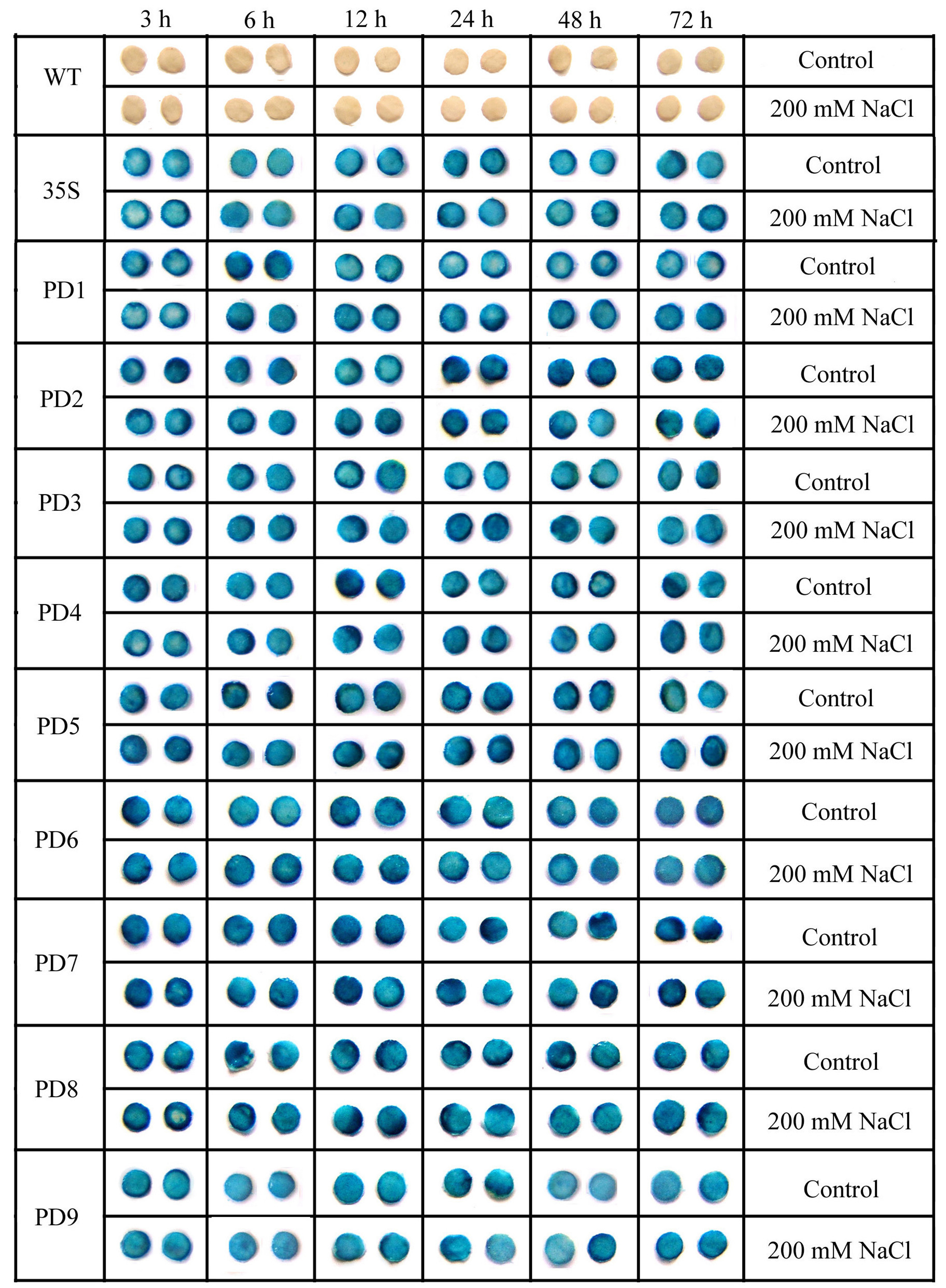

Supplement: Supplementary file 5 — Figure S4. GUS staining of detached leaves of Nicotiana benthamiana transgenic plants under normal and salt-stress conditions. Ninety leaf discs (diameter 0.5 cm) from fifteen 60-day-old individual plants (5 individual plants/ line, 3 lines for each construct) of PD1-PD9 and CaMV35S transgenic plants were incubated in liquid 1/2 MS medium supplemented with 200 mM NaCl at 25 °C for 3, 6, 12, 24, 48, and 72 h; leaf discs floated in liquid 1/2 MS medium were used as control. The leaf discs of PD1-PD9 and CaMV35S transgenic plants were then incubated in staining solution at 37 °C for 3 h. Finally, the samples were observed and photographed after decolorization. (JPG 3572 kb) [file 12896_2018_470_MOESM5_ESM.jpg]

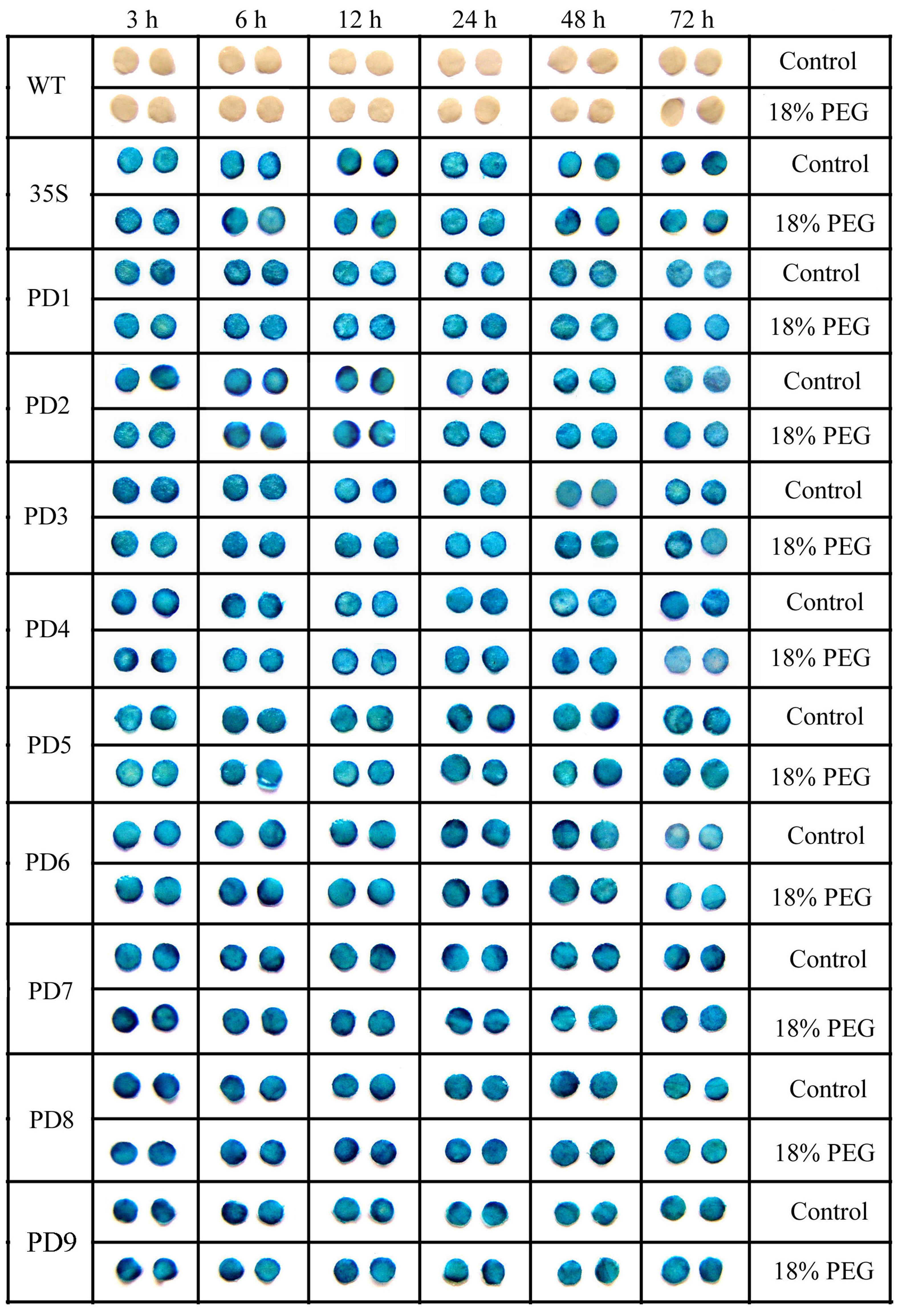

Supplement: Supplementary file 6 — Figure S5. GUS staining of detached leaves of Nicotiana benthamiana transgenic plants under normal and PEG treatment conditions. Ninety leaf discs (diameter 0.5 cm) from fifteen 60-day-old individual plants (5 individual plants/line, 3 lines for each construct) of PD1-PD9 and CaMV35S transgenic plants were incubated in liquid 1/2 MS medium supplemented with 18% PEG6000 (w/v) at 25 °C for 3, 6, 12, 24, 48, and 72 h; leaf discs floated in liquid 1/2 MS medium were used as control. The leaf discs of PD1-PD9 and CaMV35S transgenic plants were then incubated in staining solution at 37 °C for 3 h. Finally, the samples were observed and photographed after decolorization. (JPG 2139 kb) [file 12896_2018_470_MOESM6_ESM.jpg]

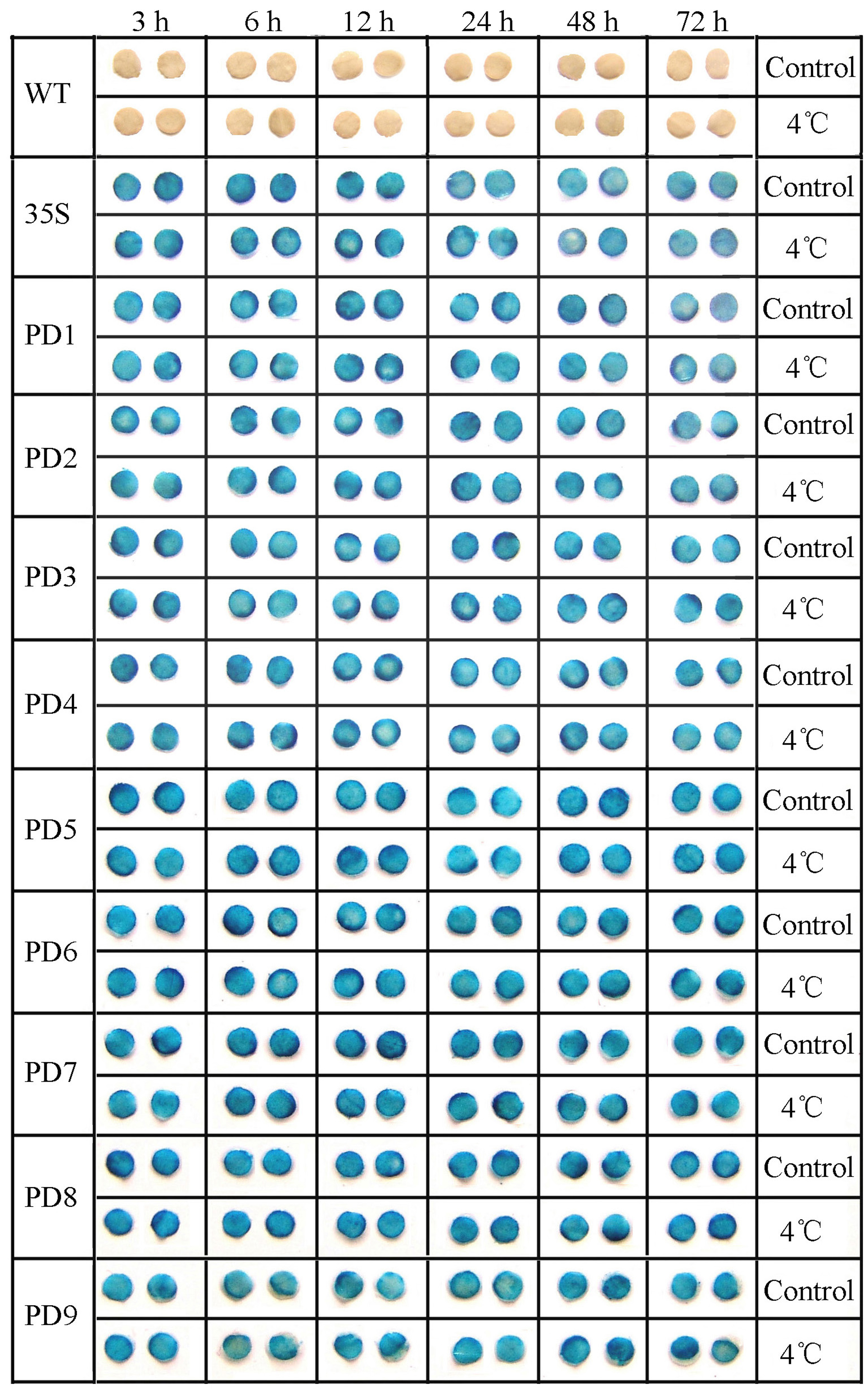

Supplement: Supplementary file 7 — Figure S6. GUS staining of detached leaves of Nicotiana benthamiana transgenic plants under normal and low-temperature conditions. Ninety leaf discs (diameter 0.5 cm) from fifteen 60-day-old individual plants (5 individual plants/line, 3 lines for each construct) of PD1-PD9 and CaMV35S transgenic plants were incubated in liquid 1/2 MS medium at 4 °C for 3, 6, 12, 24, 48, and 72 h; leaf discs floated in liquid 1/2 MS medium at 25 °C were used as control. The leaf discs of PD1-PD9 and CaMV35S transgenic plants were then incubated in staining solution at 37 °C for 3 h. Finally, the samples were observed and photographed after decolorization. (JPG 2225 kb) [file 12896_2018_470_MOESM7_ESM.jpg]
